# A stochastic programming approach for EOL electric vehicle batteries recovery network design under uncertain conditions

**DOI:** 10.1038/s41598-024-51169-6

**Published:** 2024-01-09

**Authors:** Wei Yan, Xiao Wang, Ying Liu, Xu-mei Zhang, Zhi-gang Jiang, Lin Huang

**Affiliations:** 1 School of Automotive and Traffic Engineering, Wuhan University of Science and Technology, Hubei, 430081 China; 2 Academy of Green Manufacturing Engineering, Wuhan University of Science and Technology, Hubei, 430081 China; 3 Department of Mechanical Engineering, School of Engineering, Cardiff University, Cardiff, CF24 3AA UK

**Keywords:** Environmental sciences, Environmental social sciences, Energy science and technology, Engineering

## Abstract

With the development of the electric vehicle industry, the number of power batteries has increased dramatically. Establishing a recycling EOL (end-of-life) battery network for secondary use is an effective way to solve resource shortage and environmental pollution. However, existing networks are challenging due to the high uncertainty of EOL batteries, e.g., quantity and quality, resulting in a low recycling rate of the recovery network. To fill this gap, this paper proposes a stochastic programming approach for recovery network design under uncertain conditions of EOL batteries. Firstly, a multi-objective model for battery recovery network is established, considering carbon emissions and economic benefits. Secondly, a stochastic programming approach is proposed to clarify the model. Subsequently, the genetic algorithm is employed to solve the proposed model. Finally, a recovery network case of Region T is given to verify the credibility and superiority of the proposed method. The results demonstrate that the proposed model reduces carbon emissions by 20 metric tons and increases overall economic benefits by 10 million yuan in Region T compared to the deterministic model. Furthermore, the two portions affecting the optimization results are also discussed to provide a reference for reducing carbon emissions and improving economic efficiency in recycling networks.

## Introduction

In response to the global energy crisis and the imperative to strengthen environmental protection, the National Development and Reform Commission (NDRC) formulated the “New Energy Automobile Production Admittance Management Rule” to promote the marketization of new energy vehicles^1^. As a result, new electric vehicles (EVs), serving as an alternative to traditional vehicles, are experiencing rapid development^2^. According to a report released by the International Energy Agency (IEA), global sales of EVs increase by 55% to over 10 million in 2022. And the share of EVs in total vehicle sales rises to 14%, making its highest historical proportion^3^. In such a case, the number of batteries, serving as the core components of EVs, is on the rise^4^. As outlined in China's 14th Five-Year Plan, notable enhancements have been made in the technical indicators of energy batteries, resulting in an extension of battery service life from 8–10 to 20 years^5^. Consequently, a substantial quantity of batteries is anticipated to reach their end-of-life (EOL) in the coming decade. It is predicted that the number of EOL electric vehicle batteries is expected to expand to around 7.08 million tons, and the EV batteries recycling market may even exceed 100 billion yuan by 2030^6^. Furthermore, EV batteries contain metals such as cobalt, nickel, manganese, and lithium, possessing significant recoverable value. If these metals are hydrolyzed, they will cause acidification of the ground and produce toxins and other harmful substances^7,8^. Failure to promptly dispose of EOL batteries leads to both resource wastage and environmental harm. Therefore, an effective recovery network of EOL batteries is the key enabler for the sustainable development of the EV industry.

However, there are some unavoidable uncertainties in the battery recycling process. The battery recycling process involves several steps and statistics indicate that irregularities in the operation and management of these steps can cause uncertainties in the quantity and quality of batteries recycled. For instance, there is some risk of leakage or damage during logistics and transportation as well as a lack of standardization in the battery recycling and disposal processes. Simultaneously, there are problems of insufficient regulation and enforcement in some areas, such as illegal dumping or the re-entry of untreated batteries into the market. Additionally, the inability of customers to replace EV batteries on time can result in a diversity of battery capacity at the EOL stage. The uncertainties in the recycling process may affect the location and number of network nodes. A well-functioning recovery network is a crucial component of a used battery recycling strategy, as it reduces costs, minimizes environmental impact, maximizes profits, and enhances resource efficiency. Therefore, it is crucial to take into account the uncertainty involved in the process of recycling batteries at the end of their life cycle, and then optimize the network accordingly.

The objective of this paper is to address this gap through the design of a recycling network for EOL batteries. We examine the quality variations among EOL batteries and incorporate the secondary energy storage echelon utilization of batteries, along with the recycling of precious metal materials. Additionally, under the policies of "carbon neutrality" and "peak carbon dioxide emissions", and given the requirements of the global low-carbon strategy, we also fully consider the economic and environmental benefits of the entire logistics network. To be closer to the actual situation, we have also considered the uncertainty related to the quantity and quality of batteries in the recycling process.

Thus, this paper proposes a stochastic programming approach for recovery network design under uncertain conditions of EOL batteries. The study can effectively deal with the quantity and quality uncertainty of EOL batteries, thereby enhancing recycling efficiency. The primary contributions of this paper include:

1. This paper presents a multi-objective optimization model for EOL battery networks, taking into account uncertainties, which is not only a theoretical attempt but more importantly fits the real-world scenarios. It not only facilitates resource conservation and environmental protection but also promotes the adoption and application of sustainable energy.
2. This paper employs a blend of stochastic programming methods and triangular fuzzy numbers to address the uncertainties in both quantity and quality during the battery recycling process. Intelligent algorithms are employed to tackle this intricate problem. It not only expands the scope of the solution, but also improves the feasibility of solving the problem and its practical applicability.
3. The model is validated through a real case and the impact of uncertainties on the results is analyzed. In addition, the paper uses the actual distance of the nodes in the case identified in the AutoNavi to estimate the actual transportation costs.

The rest of this paper is organized as follows. Section “Review” reviews the relevant literature. Section “Problem statement” describes the battery recycling network and related assumptions and parameters. Section “Methodology” presents the constructed stochastic programming fuzzy model. And in this section, triangular fuzzy numbers are introduced to make the model clear and easy to solve with the help of a fuzzy chance-constrained programming model. Section “Case study” validates the model through a real-life example. The research is ended in Sect. 6 with a conclusion and recommendations based on the analysis of the results.

## Review

### Reverse logistics network design

The significance of reverse logistics extends beyond cost reduction; it encompasses enhancing resource utilization and environmental protection. In most of the literature studies, it aims to portray real life constraints in a more refined way by modeling the targets. For example, Kilic et al.^9^ developed a cost-conscious reverse logistics network work for waste electrical and electronic equipment (WEEE) considering the constraints of the recycling rate of WEEE; Alumur et al.^10^ proposed a profit maximizing modeling framework for reverse logistics network design and finally applied it to a realistic case of washing machines and tumble dryers in Germany; Sasikumar^11^ developed a mixed-integer nonlinear programming model for Indian tires that considered maximizing the profit of the reverse logistics network; Momenitabar and Mattson et al.^12^ proposed a multi-objective mixed-integer linear programming model and used it in a logistics network in the tire industry, which successfully reduced the total cost of ownership and promoted the sustainability of the supply chain; Al-Salem et al.^13^ used an optimization model for minimizing the total cost of the reverse logistics network and treated the nonlinear objective as linear by segmentation method, and the results showed that a rational reverse logistics network can achieve cost savings.

While numerous studies exist on reverse logistics network designs for various objects, the number of studies focusing directly on EOL electric vehicle batteries is limited. In the early days, Yoda et al.^14^ argued that the combustion of fossil fuels can have adverse effects on the environment. Consequently, the recycling of efficient and long-lasting batteries holds significant advantages for both environmental preservation and energy sustainability. And Beer et al.^15^ explored the potential opportunities of using EOL electric vehicle batteries for secondary applications and concluded that centralized storage and reuse of EOL batteries could create significant economic value. Therefore, effective recycling of EOL batteries can bring economic as well as environmental benefits. More and more scholars have begun to focus on the topic of building a reverse logistics network for power batteries. Among them, Kannan et al.^16^ established a multi-cycle and multi-product battery reverse logistics network model; Tadaros et al.^17^ developed a mixed-integer programming model considering the total cost of the network and applied it to the recycling of used lithium-ion batteries in Sweden. However, from a circular economy and environmental point of view, the degree of environmental impact needs to be taken into account in the optimization objectives as well. Later, through a comparative exploration of greenhouse gas emissions from the cradle-to-gate process of ordinary internal combustion engines and EVs^18^ and an estimation of greenhouse gas emissions from EV batteries^19^, it was found that carbon emissions generated during the life cycle of EVs or EV batteries are not negligible. Therefore, many articles indicate the extent of the environmental impact by calculating the carbon emissions generated during the recycling process. In exploring ways to reduce environmental impact, many scholars have found that effective recycling and remanufacturing strategies will reduce carbon emissions to some extent: Xiong et al.^20^ found that recycling and remanufacturing EOL batteries could lower greenhouse gas emissions by 6.62%; Hao et al.^21^ learned from their study that recycling can reduce greenhouse gas emissions by about 10%. Therefore, Chen et al.^22^ argued that carbon dioxide emissions should be prioritized in a closed-loop supply chain, and thus proposed a model with a dual objective of total cost and carbon emissions; Wang et al.^6^ constructed a recovery network considering remanufacturing strategies for batteries and built a model that considered both total costs and carbon emissions; Wang et al.^23^ think that reverse logistics should not only consider the economic benefits but also focus on environmental protection. To this end, they have developed a reverse logistics network optimization model for waste batteries, with the aim of reducing logistics costs and CO2 emissions; M. Momenitabar et al.^24^ designed a sustainable bioethanol supply chain network considering costs, environmental impacts, and employment opportunities, and finally used machine learning methods and meta-heuristic algorithms for solving the problem. Although carbon emissions have been a significant concern, many papers generally consider only the carbon emissions and costs during transportation and processing in the battery recycling network optimization process, and few of them also consider the echelon utilization of EOL batteries after recycling. Considering that batteries also generate carbon emissions as well as profitability issues in the reuse process, this paper considers the costs, profits, and carbon emissions in the battery recycling process from consumers to the battery echelon utilization process for secondary energy storage in building the model.

### Uncertainty in reverse logistics network optimization

Compared to models of forward logistics, there is a higher level of uncertainty in the recycling process of the product^25^. In other words, uncertainty is an inherent characteristic of reverse logistics (RL). Uncertainty could be generally found in market demand, the capacity of the facility to process the products, the return quantity of the recycled products, the quality variability of the return flow, and the associated cost parameters^26^. In 2021, Wang et al.^27^ investigated a reverse logistics network dedicated to household hazardous waste, considered a mixed integer deterministic and stochastic model, and set the objective function in the model to be an expectation value; Amin et al.^28^ designed a closed-loop supply chain network for remanufacturing tires and solved the problem of uncertainty in the demand and return of the tires by using a decision tree approach; Momenitabar and Mattson et al.^12^ proposed a fuzzy multi-objective hybrid linear programming model for the tire industry and solved the model using fuzzy robust optimization method as well as goal programming method. An extensive reading of the literature reveals that there are still relatively few articles on the impact of uncertainty in recycling on reverse logistics siting models in the EVB industry. Bao et al.^29^ considered the total cost of a reverse logistics network for retired power batteries and developed a fuzzy programming model considering the uncertainty in the percentage of end-of-life, so as to determine the location of the network facilities; Jafari and Abharian^30^ proposed a mathematical model for solving the closed-loop supply chain site selection problem in the automotive battery industry under uncertainty in recycling rate, facility capacity, and cost, and minimized the cost and impact on the environment by introducing trapezoidal fuzzy numbers.

For reverse logistics, uncertainty on the input side can complicate the structure of the network and create unmeasured cost issues. Certainly, there is always uncertainty in the quantity and quality of the batteries to be recycled. This paper explores recovery networks dealing with uncertainties in both the quality and quantity of recycled EOL batteries. And yet, fuzzy programming is highly subjective, requires iterative adjustments, and has many limitations in practice. And the results of robust optimization are too pessimistic and too rigid conservative. Meanwhile, Liang^31^ states that triangular membership functions are prone to deviations from the central value and are relatively flexible. Hence, this paper chooses to convert the constructed model into a fuzzy chance-constrained programming model based on stochastic programming. Accordingly, the article uses the triangular membership functions to represent the fuzzy numbers in the constraints and objectives, enabling it to be combined with a fuzzy chance-constrained programming model to transform the uncertainty problem into an equivalent deterministic programming model.

### A brief summary

From the literature, scholars have increasingly turned their attention to the economic and environmental benefits of EOL battery recovery, as demonstrated in Table 1. Some of the recovery papers about EOL batteries have been categorized in Table 1 based on research target, reverse network, objective function, whether echelon utilization is considered, whether a specific scenario about echelon utilization is considered for reused batteries and uncertainty modeling approach. However, it also faces several challenges.

**Table 1:** Summary of previous studies.

References	Research target	Reverse neteork	Obj.function			Echelon utilization		Uncertainty modelling approach
			Economic benefits	Economic costs	Carbon emissions	Considered	Specific to a scenario	
Kilic et al.^9^	WEEE	√	√	√				
Bao et al.^29^	Power battery	√		√		√		Fuzzy programming + triangular fuzzy number
Tadaros M et al.^17^	Lithium-ion batteries	√		√				
Sasikumar^11^	Truck tire	√	√	√				
Wang et al.^23^	Waste batteries	√		√	√			
Alumur et al.^10^	Washing machines and tumble dryers	√	√	√				
Momenitabar et al.^24^	Bioethanol	√				√	√	
Al-Salem et al.^13^	Closed-loop supply chain for a single product	√				√		
Beer et al.^15^	PEV batteries		√	√				
Kannan et al.^16^	Spent lead–acid batteries	√		√				
Xiong et al.^20^	Lithium-ion batteries				√			
Chen et al.^22^	The solar energy industry	√		√	√			
Wang et al.^6^	Electric vehicle battery	√		√	√			
Wang et al.^27^	Hazardous wastes: household paint	√		√				Two-stage stochastic programming model
Amin et al.^28^	Tire	√	√	√				Decision tree
Jafari et al.^30^	The car battery industry	√		√	√			Lexicography method + trapezoid fuzzy number
Liang^31^	The Daya ball screw plant	√	√	√				A fuzzy linear programming approach based on the possibility theory + triangular fuzzy number
Momenitabar and Mattson et al.^12^	The tire industry	√		√	√			Fuzzy robust optimization approach + fuzzy triangular numbers

A fundamental challenge lies in the high degree of uncertainty regarding the quantity and quality of EOL batteries. Existing networks struggle to harness their full potential due to this uncertainty, which affects factors such as location decisions and distribution planning. Furthermore, many EOL batteries can undergo secondary energy storage after recycling, creating additional economic benefits.

In summary, while numerous researchers have started to focus on EOL battery recycling and the uncertainties within reverse logistics networks, literature that comprehensively addresses uncertainties in the EOL battery recycling process, taking into account factors such as economic benefits, costs, carbon emissions, and the potential for secondary battery usage after recycling, remains limited. Therefore, this paper, recognizing the potential for echelon utilization of EOL batteries, establishes a multi-objective model for designing economically and environmentally efficient recycling networks. It proposes a stochastic programming approach and employs triangular fuzzy numbers to address uncertainty within the problem.

## Problem statement

### Description of the recovery network

**Figure 1:**
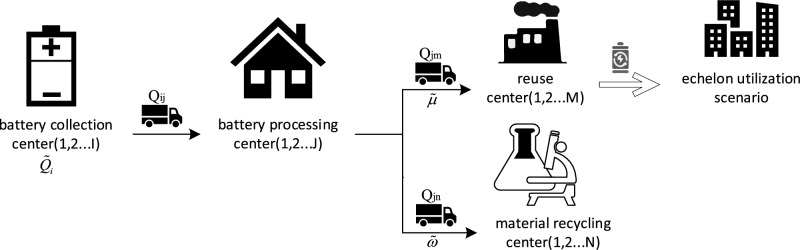
EOL batteries recovery process.

The EOL batteries recovery network includes a battery collection center, battery processing center, reuse center, and material recycling center, as shown in Fig. 1.

In order to recycle EOL batteries more efficiently, the proposed model would purchase the batteries voluntarily recovered by consumers. The battery collection center is the place where the EOL batteries are traded and is responsible for sending them to the battery processing center. And the battery processing center needs to test and screen the quality of the batteries shipped to determine whether they can continue to be used. Furthermore, the EOL batteries eligible for continued use can be reassembled in new batteries, thereby being utilized in other scenarios. Otherwise, the EOL batteries will be sent to a material recycling center for deep dismantling and recycling of heavy metals. The proposed model will be able to determine the location of every type of center constructed in this network and the quantity of EOL batteries which be flowed from one center to another.

### Main modeling assumptions

The proposed model has ten constraints. To simplify the model, the following assumptions are proposed:

- The flow of the EOL batteries only occurs between different centers, and it does not flow between centers of the same kind.
- The price of EOL batteries in the battery collection center is the same. Each center’s construction cost is fixed.
- The locations of centers are uncertain, but the number of alternative centers and the capacity constraints of centers are known in advance.
- Battery collection centers have no time limits.
- According to the NGGI (National Greenhouse Gas Inventories) from IPCC (Intergovernmental Panel on Climate Change), only the CO2 emissions in the network are taken into consideration^32^.

### Notation

In this paper, we introduce the notation, encompassing sets, parameters, decision variables, and fuzzy variables used to design the recovery network. In order to achieve the desired results, determining the accurate values of the notations is crucial. Therefore, within the limits of the mathematical model, the values of the following main notations will be determined in List of symbols section.

## Methodology

In this section, the paper constructs a stochastic programming-based recovery network model for EOL batteries. First, the stochastic programming approach will be introduced, followed by a description of the model constructed. Then, the existing model will be converted into a deterministic model. Finally, the model-solving method will be identified.

### Stochastic programming

Stochastic programming is a frequently used method of optimization under uncertainty. As it can be seen in the above literature, robust optimization and stochastic programming are two common optimization methods under uncertainty. Due to the intractability and higher costs of robust optimization^33^ and the assumption that the probability distribution of uncertain parameters is known in stochastic programming as proposed by Dantzig (1955)^34^, the more tractable stochastic programming method specifically designed to deal with modeling problems involving uncertainty was chosen for this paper^35^. In this paper, a fuzzy chance-constrained programming model in stochastic programming is introduced.

### Model construction

The paper expresses the uncertainty of the quantity of EOL batteries recycled in terms of its ambiguity, using the ambiguity of the probability of reuse and material recycling to express the uncertainty of the quality of EOL batteries recycled.

#### Objective function

The model encompasses two objectives. According to the above assumptions, a multi-objective model is developed to optimize site selection in the entire recovery network to minimize carbon emissions and maximize the economic benefits of the whole network.

1 $$MAX \, F = F1 + F2 - F3 - F4 - F5 - F6$$

2 $$MIN \, P = P1 + P2 + P3 + P4$$

where Eqs. (1) and (2) represent the objective function of the model. As shown below, the economic benefits of the entire network comprise six parts, i.e., benefits from batteries used for echelon utilization and material recycling F1; government subsidy F2; fixed cost F3; inter-center transportation cost F4; processing cost F5; and purchase cost F6. In Eq. (2), CO2 emissions are summed from inter-center transportation, the center facility establishment, processing, and echelon utilization scenarios, which are represented by P1, P2, P3, and P4.

##### Economic benefits

The economic benefit from reuse and material recycling in Eq. (3) refers to the fact that after recycled EOL batteries, they are usually sent to reuse centers or material recycling centers for processing in order to realize their residual value. In general, the echelon utilization scenario for recycled EOL batteries is battery energy storage system (BESS), based on Ref.^36–40^. This paper considered the benefit generated by the energy storage scenario over the life cycle, not limited to the first year. Therefore, EOL batteries that can continue to be used will be sent to the energy storage system for energy storage to create income. EOL batteries that cannot be reused in the network will be sent to a material recycling center for precious metal recycling.

3 $$F1 = \sum\limits_{{}} {p \cdot Q} = \sum\limits_{i \in I} {\sum\limits_{j \in J} {\left( {p_{11} Q_{ij} \tilde{\mu } + p_{n} Q_{ij} \tilde{\omega }} \right)} }$$

Government subsidy means that as the government attaches importance to the recycling of EOL batteries, the state will introduce relevant policies to achieve compensation for the recycling of unit batteries, in Eq. (4).

4 $$F2 = g\sum\limits_{i \in I} {\tilde{Q}_{i} }$$

The fixed construction cost refers to the fixed cost needed to build the processing center, reuse center, and material recycling center of the EVB recovery network, in Eq. (5).

5 $$F3 = \sum {y \cdot f = } \sum\limits_{j \in J} {y_{j} f_{j} + \sum\limits_{m \in M} {y_{m} } f_{m} + \sum\limits_{n \in N} {y_{n} } f_{n} }$$

Transport cost refers to the cost incurred in transporting the recycled EOL batteries between the various centers of the network, in Eq. (6).

6 $$F4 = \sum\limits_{{}} {Q \cdot d \cdot c} = \sum\limits_{i \in I} {\sum\limits_{j \in J} {\left( {Q_{ij} d_{ij} c + \sum\limits_{m \in M} {Q_{ij} \tilde{\mu }} d_{jm} c + \sum\limits_{n \in N} {Q_{ij} \tilde{\omega }d_{jn} c} } \right)} }$$

The processing cost refers to the cost incurred by each center in processing recycled EOL batteries. The processing cost of each center is calculated by Eq. (7).

7 $$F5 = \sum\limits_{{}} {Q \cdot c} = \sum\limits_{i \in I} {\sum\limits_{j \in J} {\left( {Q_{ij} c_{j} + \sum\limits_{m \in M} {Q_{ij} } \tilde{\mu }c_{m} + \sum\limits_{n \in N} {Q_{ij} } \tilde{\omega }c_{n} } \right)} }$$

The purchase cost refers to the recycling cost of the EOL batteries from the consumption area in the network, in Eq. (8). It means that the recycling of EOL batteries can be promoted by providing subsidies to consumers.

8 $$F6 = p\sum\limits_{i \in I} {\tilde{Q}_{i} }$$

In this paper, the first objective is to maximize the total economic benefits of the network of EOL batteries for EVs, and the following formula is established.

9 $$\begin{gathered} MAX \, F = F1 + F2 - F3 - F4 - F5 - F6 \\ = \sum\limits_{i \in I} {\sum\limits_{j \in J} {\left( {p_{11} Q_{ij} \tilde{\mu } + p_{n} Q_{ij} \tilde{\omega }} \right)} } + g\sum\limits_{i \in I} {\tilde{Q}_{i} } - \left( {\sum\limits_{j \in J} {y_{j} f_{j} + \sum\limits_{m \in M} {y_{m} } f_{m} + \sum\limits_{n \in N} {y_{n} } f_{n} } } \right) \\ - \sum\limits_{i \in I} {\sum\limits_{j \in J} {\left( {Q_{ij} d_{ij} c + \sum\limits_{m \in M} {Q_{ij} \tilde{\mu }} d_{jm} c + \sum\limits_{n \in N} {Q_{ij} \tilde{\omega }d_{jn} c} } \right)} } \\ - \sum\limits_{i \in I} {\sum\limits_{j \in J} {\left( {Q_{ij} c_{j} + \sum\limits_{m \in M} {Q_{ij} } \tilde{\mu }c_{m} + \sum\limits_{n \in N} {Q_{ij} } \tilde{\omega }c_{n} } \right)} } - p\sum\limits_{i \in I} {\tilde{Q}_{i} } \\ \end{gathered}$$

##### Carbon emissions

Environmental costs are generally calculated by monitoring greenhouse gases in the environment, including CO2 generated during the transportation and processing of batteries related to the network. Furthermore, this paper takes into account the carbon emissions produced by the reuse of batteries.

P1 refers to the carbon emissions from transportation in the network.

10 $$P1 = a\sum\limits_{{}} {d \cdot Q} = a\sum\limits_{i \in I} {\sum\limits_{j \in J} {\left( {d_{ij} Q_{ij} + \sum\limits_{m \in M} {d_{jm} Q_{ij} \tilde{\mu } + } \sum\limits_{n \in N} {d_{jn} Q_{ij} } \tilde{\omega }} \right)} }$$

P2 refers to the carbon emissions from the process of center construction.

11 $$P2 = by_{j} + by_{m} + by_{n}$$

P3 refers to the carbon emissions from each center in the processing of the battery.

12 $$P3 = \sum\limits_{{}} {e \cdot Q} = \sum\limits_{i \in I} {\sum\limits_{j \in J} {\left( {e_{j} Q_{ij} + \sum\limits_{n \in N} {e_{n} Q_{ij} } \tilde{\mu } + \sum\limits_{m \in M} {e_{m} Q_{ij} } \tilde{\omega }} \right)} }$$

P4 refers to the carbon emissions from the Echelon utilization scenario.

13 $$P4 = \beta \cdot Q_{jm}$$

The second objective is to minimize the total carbon emissions of the network of EOL batteries for EVs.

14 $$\begin{gathered} MIN \, P = P1 + P2 + P3 + P4 \\ = a\sum\limits_{i \in I} {\sum\limits_{j \in J} {\left( {d_{ij} Q_{ij} + \sum\limits_{m \in M} {d_{jm} Q_{ij} \tilde{\mu } + } \sum\limits_{n \in N} {d_{jn} Q_{ij} } \tilde{\omega }} \right)} } + \left( {by_{j} + by_{m} + by_{n} } \right) \\ + \sum\limits_{i \in I} {\sum\limits_{j \in J} {\left( {e_{j} Q_{ij} + \sum\limits_{n \in N} {e_{n} Q_{ij} \tilde{\mu }} + \sum\limits_{m \in M} {e_{m} Q_{ij} } \tilde{\omega }} \right)} } + \beta \cdot Q_{jm} \\ \end{gathered}$$

#### Constraints

To solve the model, the following constraints are considered.

##### Demand constraints

The demand for batteries at collection centers is limited. Constraint (15) indicates the demand restriction constraints of the collection centers.

15 $$\tilde{Q}_{i} = \sum\limits_{j \in J} {Q_{ij} } y_{j} \, i = 1,2,3...I$$

##### Mass balance

The EOL battery flow of centers is balanced in the recovery network. Constraints (16) represent the mass equilibrium of the battery processing centers.

16 $$\sum\limits_{i \in I} {Q_{ij} } = \tilde{\mu }\sum\limits_{i \in I} {Q_{ij} } y_{j} + \tilde{\omega }\sum\limits_{i \in I} {Q_{ij} } y_{j} \, j = 1,2,3...J$$

##### Capacity constraint

The processing capacity of the battery processing center, the remanufacturing capacity of the reuse center, and the recycling capacity of the material recycling center are limited. Constraints (17) represent restrictions on the battery processing center, reuse center, and material recycling center.

17 $$0 \le \sum\limits_{{}} {Q_{{}} } \le Max \cdot y_{{}} \,$$

##### Decision variables constraints

Constraints (18) are related to the corresponding decision variables.

18 $$y_{j} ,y_{m} ,y_{n} = \left\{ {\begin{array}{*{20}c} {1,} & {if \, the \, center \, j \in J,\,m \in M,\,n \in N} \\ {0,} & {otherwise} \\ \end{array} } \right.$$

##### Other constraints

Constraint (19) indicates that the sum of the proportion of batteries shipped to the reuse center and material recycling center is 1.

19 $$\tilde{\mu } + \tilde{\omega } = 1$$

### Model transformation

Survey statistics show that the carbon tax is an essential tool to promote remanufacturing and reduce carbon emissions^41^. According to the carbon tax policy, the processes associated with carbon emissions will involve the cost of the carbon tax^42^. As a result, the article combines the multi-objective optimization problem with the carbon tax, treating carbon emissions as environmental costs and converting the model into an easily solvable single objective function. As the above model contains related parameters of recycling quantity and recycling quality $\tilde{Q}_{i} , \, \tilde{\mu }, \, \tilde{\omega }$, it cannot be solved directly. The paper applies stochastic programming theory and triangular fuzzy numbers to convert the model into a deterministic one. The triangular fuzzy numbers used in this paper is one of the most commonly used fuzzy numbers^43^, which can more accurately express the fuzziness and uncertainty of complex data.

The fuzzy chance-constrained programming mentioned above is a kind of uncertain mathematical planning based on possibility theory and fuzzy set theory, which means that when there are fuzzy parameters in the decision environment, the decision result may not satisfy the constraint limit under certain circumstances. In order to make the objective function and the constraints can meet the constraints, it is necessary to set a certain confidence level, that is, solving for the optimal solution of the model under certain probability conditions.

Typically, the fuzzy chance-constrained programming model with fuzzy parameters is used for modeling as follows:

$$\left\{ \begin{gathered} \min \overline{f}(x,\xi ) \hfill \\ st. \hfill \\ pos[f(x,\xi ) \le \overline{f}] \ge \partial \hfill \\ pos[g_{i} (x,\xi ) \le 0] \ge \beta_{i} \, i = 1,2...I \hfill \\ \end{gathered} \right.,$$

where $x$ is the decision variable; $\xi$ is the fuzzy covariate; $f$ is the objective function; $g$ is the constraint function; $\partial$ is the confidence level of the objective function; $\beta$ is the confidence level of the *i*th constraint; $pos[ \cdot ]$ denotes the probability of the event.

The key to solving fuzzy chance-constrained optimal problems is to deal with chance constraints. One method is fuzzy simulation, but the solution process is time-consuming and imprecise. Another approach is to convert the chance constraint into a clear equivalence class and then solve the model. Given the uncertainty of the objective situation and the vagueness of human thinking, fuzzy set theory has become the main method for decision making. In particular, triangular fuzzy numbers or fuzzy languages have been widely used in fuzzy control and decision making^44,45^. For example, Wang et al.^46^ proposed a comprehensive evaluation method for group support systems using triangular fuzzy number measurement indicators. Thus, in order to simplify the process of solving the model, the paper introduces triangular fuzzy numbers to clarify the fuzzy chance constraints.

**Figure 2:**
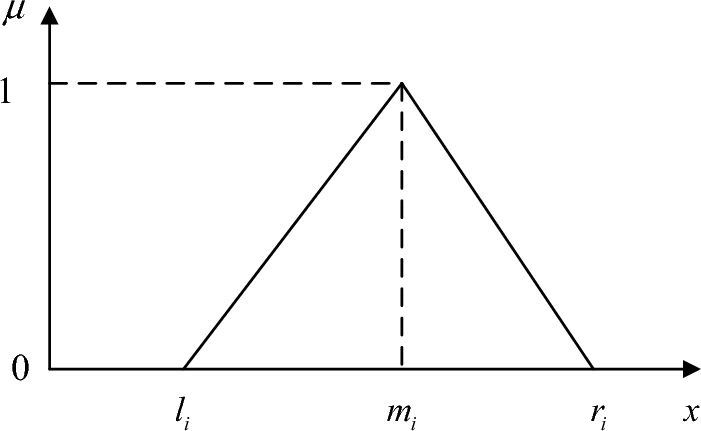
The image of membership function.

Assume that the triangular fuzzy number is $\tilde{\partial } = (l_{i} ,m_{i} ,r_{i} )$, and the membership function is $\mu (x)$, then: $$\left\{ \begin{gathered} pos(\tilde{\partial }_{i} \le z) = \sup \left\{ {\left. {\mu_{{\tilde{d}}} (x|x \in R,z \ge x)} \right\}} \right. \hfill \\ pos(\tilde{\partial }_{i} \ge z) = \sup \left\{ {\left. {\mu_{{\tilde{d}}} (x|x \in R,z \le x)} \right\}} \right. \hfill \\ pos(\tilde{\partial }_{i} = z) = \mu_{{\tilde{d}}} (z) \hfill \\ \end{gathered} \right.$$. For any given confidence level $\partial (0 \le \partial \le 1)$, $pos(\tilde{\partial } \le z) \ge \partial$ is valid if and only if the conditions for $(1 - \partial )l_{i} + \partial m_{i} \le z$ is satisfied; for any given confidence level $\beta (0 \le \beta \le 1)$, $pos(\tilde{\partial } = z) \ge \beta$ is valid if and only if the conditions for $$\left\{ {\begin{array}{*{20}c} {(1 - \beta )l_{i} + \beta m_{i} \ge z} \\ {(1 - \beta )r_{i} + \beta m_{i} \le z} \\ \end{array} } \right.$$ are satisfied. Where $m_{i}$ is called the central value of $\partial$, $l_{i}$ and $r_{i}$ are the left and right boundaries of $\partial$. When $\partial = l_{i} = m_{i} = r_{i}$, $\partial$ is an exact value. The membership function $\mu$ ^47^represents the likelihood measure of the fuzzy factor and can be defined as $$\mu_{{\tilde{a}}} (x) = \left\{ {\begin{array}{*{20}c} 0 \\ {\frac{{x - l_{i} }}{{m_{i} - l_{i} }}} \\ {\frac{{r_{i} - x}}{{r_{i} - m_{i} }}} \\ 0 \\ \end{array} } \right. \, \begin{array}{*{20}c} {x < l_{i} } \\ {} \\ {l_{i} \le x < m_{i} } \\ {} \\ {m_{i} \le x < r_{i} } \\ {x \ge r_{i} } \\ \end{array}$$, the image of membership function is shown in Fig. 2.

According to the above model, the transformed fuzzy chance-constrained programming model is as follows:

20 $$MIN \, Z^{\prime} = pos\{ - F1 - F2 + F3 + F4 + F4 + F5 + F6 + h(P1 + P2 + P3 + P4) \le \overline{Z}_{1} \} \ge \partial_{1}$$

St.

21 $$pos\left\{ {\left. {\tilde{Q}_{i} = \sum\limits_{j \in J} {Q_{ij} } y_{j} } \right\}} \right. \ge \beta_{1} \, i = 1,2,3...I$$

22 $$pos\left\{ {\left. {\tilde{\mu }\sum\limits_{i \in I} {Q_{ij} } y_{j} = \sum\limits_{m \in M} {Q_{jm} } y_{m} } \right\}} \right. \ge \beta_{2} \, j = 1,2,3...J$$

23 $$pos\left\{ {\left. {\tilde{\omega }\sum\limits_{i \in I} {Q_{ij} } y_{j} = \sum\limits_{m \in M} {Q_{jn} } y_{n} } \right\}} \right. \ge \beta_{3} \, j = 1,2,3...J$$

The paper uses triangular fuzzy numbers to represent the fuzzy recycling quantity $\tilde{Q}_{i}$ and fuzzy recycling quality $\mu$ and $\tilde{\omega }$ in the model, and uses the confidence levels to limit the magnitude of risk due to uncertainty, making the above model and constraints clearer. Deriving Eqs. (20) ~ (23) gives Eqs. (24) ~ (27).

24 $$\begin{gathered} [ - \sum\limits_{i \in I} {\sum\limits_{j \in J} {(p_{11} } } Q_{ij} [(1 - \partial_{1} )\mu_{li} + \partial_{1} \mu_{mi} ] + p_{n} Q_{ij} [(1 - \partial_{1} )\omega_{li} + \partial_{1} \omega_{mi} ]) \hfill \\ - g\sum\limits_{i \in I} {[(1 - \partial_{1} )Q_{ili} + \partial_{1} Q_{imi} ]} + (\sum\limits_{j \in J} {y_{j} f_{j} + \sum\limits_{m \in M} {y_{m} } f_{m} + \sum\limits_{n \in N} {y_{n} } f_{n} } ) \hfill \\ + \sum\limits_{i \in I} {\sum\limits_{j \in J} {(Q_{ij} } } d_{ij} c + \sum\limits_{m \in M} {Q_{ij} [(1 - \partial_{1} )\mu_{li} + \partial_{1} \mu_{mi} } ]d_{jm} c + \sum\limits_{n \in N} {Q_{ij} [(1 - \partial_{1} )\omega_{li} + \partial_{1} \omega_{mi} ]d_{jn} c} ) \hfill \\ + \sum\limits_{i \in I} {\sum\limits_{j \in J} {(Q_{ij} } } c_{j} + \sum\limits_{m \in M} {Q_{ij} } [(1 - \partial_{1} )\mu_{li} + \partial_{1} \mu_{mi} ]c_{m} + \sum\limits_{n \in N} {Q_{ij} } [(1 - \partial_{1} )\omega_{li} + \partial_{1} \omega_{mi} ]c_{n} ) \hfill \\ + p\sum\limits_{i \in I} {[(1 - \partial_{1} )Q_{ili} + \partial_{1} Q_{imi} ]} \hfill \\ + h \cdot a \cdot \sum\limits_{i \in I} {\sum\limits_{j \in J} {(d_{ij} Q_{ij} } } + \sum\limits_{m \in M} {d_{jm} Q_{ij} [(1 - \partial_{2} )\mu_{li} + \partial_{2} \mu_{mi} ]} + \sum\limits_{n \in N} {d_{jn} Q_{ij} } [(1 - \partial_{2} )\omega_{li} + \partial_{2} \omega_{mi} ]) \hfill \\ + (by_{j} + by_{m} + by_{n} ) \hfill \\ + \sum\limits_{i \in I} {\sum\limits_{j \in J} {(e_{j} } } Q_{ij} + \sum\limits_{n \in N} {e_{n} Q_{ij} } [(1 - \partial_{2} )\mu_{li} + \partial_{2} \mu_{mi} ] + \sum\limits_{m \in M} {e_{m} Q_{ij} } [(1 - \partial_{2} )\omega_{li} + \partial_{2} \omega_{mi} ]) \hfill \\ + \beta \cdot Q_{jm} )] \le \overline{Z}_{1} \hfill \\ \end{gathered}$$

St.

25 $$\left\{ \begin{gathered} [(1 - \beta_{1} )Q_{ili} + \beta_{1} Q_{imi} ] \le \sum\limits_{j \in J} {Q_{ij} } y_{j} \, \hfill \\ [(1 - \beta_{1} )Q_{iri} + \beta_{1} Q_{imi} ] \ge \sum\limits_{j \in J} {Q_{ij} } y_{j} \, \hfill \\ \end{gathered} \right. \, i = 1,2,3...I \,$$

26 $$\left\{ \begin{gathered} [(1 - \beta_{2} )\mu_{li} + \beta_{2} \mu_{mi} ]\sum\limits_{i \in I} {Q_{ij} } y_{j} \le \sum\limits_{m \in M} {Q_{jm} } y_{m} \, \hfill \\ [(1 - \beta_{2} )\mu_{ri} + \beta_{2} \mu_{mi} ]\sum\limits_{i \in I} {Q_{ij} } y_{j} \ge \sum\limits_{m \in M} {Q_{jm} } y_{m} \hfill \\ \end{gathered} \right. \, j = 1,2,3...J$$

27 $$\left\{ \begin{gathered} [(1 - \beta_{3} )\omega_{li} + \beta_{3} \omega_{mi} ]\sum\limits_{i \in I} {Q_{ij} } y_{j} \le \sum\limits_{n \in N} {Q_{jn} } y_{n} \, \hfill \\ [(1 - \beta_{3} )\omega_{ri} + \beta_{3} \omega_{mi} ]\sum\limits_{i \in I} {Q_{ij} } y_{j} \ge \sum\limits_{n \in N} {Q_{jn} } y_{n} \hfill \\ \end{gathered} \right. \, j = 1,2,3... \, J$$

### Model solution

**Figure 3:**
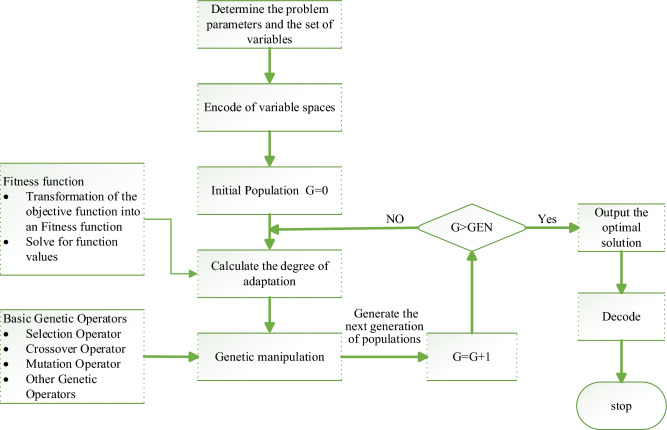
Flow-process diagram of algorithm.

The above model falls within the category of network optimization problems. In addressing such problems, conventional solving algorithms have traditionally been favored in the literature. However, these conventional methods are associated with significant computational costs, susceptibility to local optima, and low solving efficiency, as noted in reference^48^. Therefore, many scholars use intelligent algorithms, such as genetic algorithms (GA) and taboo search. Compared with traditional algorithms, intelligent algorithms have better stability. In the 1970s, the genetic algorithm was first mentioned as a technical term^49^. GA is able to find the global optimal solution rather than just the local optimal solution by searching multiple solution spaces in the problem space. Furthermore, GA uses probabilistic search methods to automatically obtain the optimal search space, which is very suitable for network optimization problems^50^ including routing problems, resource allocation problems, and scheduling problems, etc.^51^, and plays a great role in solving network optimization problems. Given the robust theoretical foundation and extensive success in both theoretical and practical applications^52^, along with GA's simplicity, ease of use, and high efficiency, this paper opts for GA as the solution for the model. The calculation steps are shown in Fig. 3.

*Step 1*: Import of basic data and encode.

Firstly, the parameters in the model and the base data of the network are imported to complete the parameter initialization. The coding method is determined according to the characteristics of the variables, and the corresponding initial populations are randomly generated by the coding operation.

*Step 2*: Population initialization.

According to the encoding method selected in Step 1, N random initial string structures are generated to form the population, each string structure is an individual of the population, and N is the size of the generated population.

*Step 3*: Calculate the fitness value.

In a genetic algorithm, the value of the fitness function is the criterion for distinguishing the goodness of individuals in a population. The fitness function is developed based on the objective function, and the population is decoded using certain rules to calculate individual fitness values.

*Step 4*: Genetic operation.

Genetic operators perform the recombination and mutation of genetic algorithms, including selection, crossover, mutation, etc. The genetic material drives the optimization of the population in these three ways, moving on to Step 2, finding the superior offspring.

*Step 5*: Determine termination conditions.

One of the reasons why a genetic algorithm is so computationally efficient is that it can set conditions to control the algorithm process, thus reducing useless computations. When the algorithm satisfies the target termination and iteration termination conditions, the operation is terminated and the optimal result is output; if not, the above steps are continued.

## Case study

### Background

**Figure 4:**
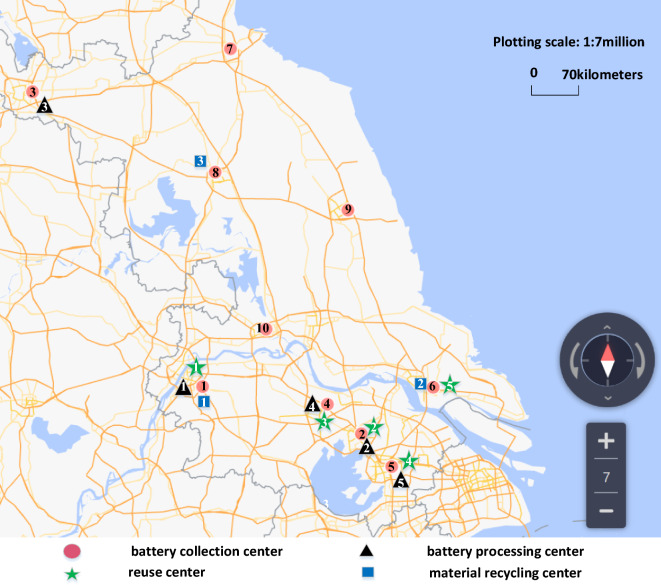
Alternative locations for each facility center. Source: Data collected from AMAP Inside (Version number: 1.0.15. URL: https://lbs.amap.com/).

The paper takes the Region T in China as an example. As a major province in the manufacture and consumption of new energy vehicles in China, new EVs in Region T are growing rapidly and contribute significantly to the national total^53^. In response to policies and regulations supporting battery recycling, and considering the uncertainty of the quantity and quality of EOL batteries in the recycling process, the paper proposes an EOL batteries recovery network. The output of Lithium iron phosphate (LFP batteries) batteries in China reached 125.4 GWh in 2021, accounting for 57.1% of the total power batteries output in China. And in 2021, the National Energy Administration (NEA) issued the “Twenty-five Key Requirements for Preventing Electricity Production Accidents”, which states that ternary lithium-ion batteries (NMC batteries) will no longer be used for energy storage. The market share of LFP batteries increased further in 2022, and its production accounted for 61% of the total production of power batteries. Therefore, this paper focuses on the recycling of LFP batteries in Region T^54^. As shown in Fig. 4, ten battery collection centers, five battery processing centers, five reuse centers, and three material recycling centers are involved in the battery recycling industry chain in Region T. In particular, the scale of the map in Fig. 4 is 1:7000,000, where 1 cm on the map corresponds to 70 km in the actual physical space.

### Date description

According to the survey statistics, it is assumed that the ratio of the batteries flowing to the reuse center and material recycling center is generally 0.88 and 0.12 based on the reference^55^. From other literature, it is known that fuzzy affiliation generally maps 10% of the number of fuzzy centers^56^. Thus, the article makes that the fuzzy affiliation maps within 10% of the number of fuzzy centers. Based on relevant information and actual enterprise application data research results, Table 2 shows the number of EOL batteries of each region, expressed in kWh^8,57^. The values are presented in the original units (kg) and for clarity, based on the calculations from the Chinese LCA Database also in kWh using the conversion 1 kWh = 8.643 kg. Other uncertain parameters involved in the network are shown in Table 3.

**Table 2:** Quantity of recycled at each collection center of Region T (unit: kWh).

Battery collection center	Recycling quantity	Triangular fuzzy number:$(l_{i} ,m_{i} ,r_{i} )$		
		$Q_{ili}$	$Q_{imi}$	$Q_{iri}$
1	41,665.96	37,499.36	41,665.96	45,832.56
2	33,960.28	30,564.25	33,960.28	37,356.31
3	30,680.66	27,612.59	30,680.66	33,748.72
4	22,508.87	20,257.98	22,508.87	24,759.76
5	55,225.73	49,703.16	55,225.73	60,748.30
6	31,298.72	28,168.85	31,298.72	34,428.59
7	14,806.41	13,325.76	14,806.41	16,287.05
8	16,934.15	15,240.74	16,934.15	18,627.57
9	24,824.53	22,342.08	24,824.53	27,306.98
10	19,485.02	17,536.52	19,485.02	21,433.52

**Table 3:** Other fuzzy parameters.

Fuzzy parameters	Triangular fuzzy number:$(l_{i} ,m_{i} ,r_{i} )$
$\tilde{\mu }$	(0.792, 0.88, 0.968)
$\tilde{\omega }$	(0.108, 0.12, 0.132)

The unit-relevant parameters are shown in Table 4. Meanwhile, according to the "Notice on the key work related to the management of enterprise greenhouse gas emission report in 2022" issued by the Ministry of Ecology and Environment of the People's Republic of China (MEEPRC), the latest grid emission factor is 0.5810tCO2/MWh^58^. As stated by the “2021 China Carbon Price survey report”, the average carbon price in the national carbon market in 2022 is expected to be 49 yuan/ton^59^.

**Table 4:** Unit parameters.

Parameters	Value
Processing cost in the processing center	17,000 yuan per metric ton^8,18^
Processing cost in reuse center	1,28,000 yuan per metric ton^8,18^
Processing cost in a material recycling center	42,000 yuan per metric ton^8^
Fixed construction costs in the processing center	0.8 million yuan^6^
Fixed construction costs in the reuse center	1 million yuan
Fixed construction costs in the material recycling center	0.6 million yuan
Unit transportation cost	0.33 yuan per metric ton per kilometer
Unit battery acquisition cost in the collection center	8000 yuan per metric ton
Unit battery government subsidy	20 yuan/kWh
Unit battery benefits in a material recycling center	30,000 yuan per metric ton

The processing center connects the collection center and reuse center, as well as the material recycling center, so its location directly affects the network. Region T can be roughly divided into 10 regions. Based on the number of logistics parks or new energy companies in each region, the corresponding region is selected as an alternative location for the battery processing centers, reuse centers, and material recycling centers. And the location coordinates of alternative centers are calculated from AutoNavi.

The related emission data and capacity limits are shown in Table 5. The paper assumes that each alternative center covers 200 square meters. Based on the relevant data and actual enterprise application data finding, the confidence level of uncertain parameters is set, in which suppose $\partial_{1} = \partial_{2} = 0.9$,$\beta_{1} = \beta_{2} = \beta_{3} = 0.8$.

**Table 5:** Other data.

	Battery processing center	Reuse center	Material recycling center
Processing capacity	1·10^3^ metric tons	8·10^2^ metric tons	2·10^2^ metric tons
Unit transportation CO2 emissions	0.0102 CO_2_-equivalent ton/ (ton·km)^60^		
Carbon emissions from construction individual center	0.5 metric ton/m^2,61^		
Carbon emissions of processing batteries in different center	0.694 CO_2_-equivalent ton per metric ton^6,62^	8.866 CO_2_-equivalent ton per metric ton^8,63–65^	2.443 CO_2_-equivalent ton per metric ton^66^

### Results and discussion

#### Results

**Figure 5:**
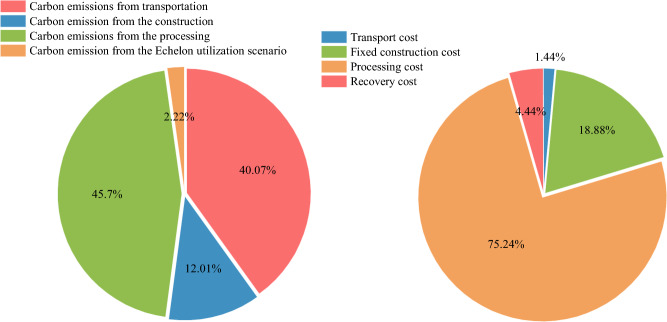
Percentage of Carbon emissions and Economic costs by category.

The paper utilized MATLAB2022Ra software to perform this algorithmic example with a standalone run time of 120 s. A laptop with Intel Core i5 processor and 16 GB RAM was used as the hardware device. The case study was solved using the Improved Hybrid Collaborative Genetic Algorithm, in which the population size was assumed to be 200, the maximum number of iterations to be 600, the maximum crossover probability to be 0.8; and the minimum crossover probability to be 0.2. When the constraint is satisfied, it can be seen from Tables 6 and 7 that the logistics network is most environmentally friendly and economically beneficial when J1, J2, and J4 are selected for the battery processing center, M1, M2, and M3 for the reuse center, and N1 and N3 for the material recycling centers. The optimal total economic benefits value of the model is calculated to be 0.229845 trillion yuan, of which the optimal environmental cost is 15,999 yuan and the economic cost is 0.457549 trillion yuan. As shown in Fig. 5, nearly 46% of total carbon emissions arise from the batteries processing process, followed by the transportation process. And the batteries processing process generates the most economic costs and found it accounted for 75% of total economic costs, followed by the transportation process.

**Table 6:** Flow Distribution from the battery collection centers to the processing centers.

Notation	J1	J2	J4
I1	41,665.96	0	0
I2	0	0	33,960.28
I3	30,680.66	0	0
I4	0	0	22,508.87
I5	0	55,225.73	0
I6	0	0	31,298.72
I7	14,806.41	0	0
I8	16,934.15	0	0
I9	0	24,824.53	0
I10	0	0	19,485.02

**Table 7:** Flow Distribution from the battery processing centers to the next centers.

Notation	M1	M2	M3	N1	N3
J1	87,153.03	0	0	16,934.15	0
J2	0	80,050.26	0	0	0
J4	0	0	87,767.87	0	19,485.02

#### Discussion

##### Comparison of model results

**Figure 6:**
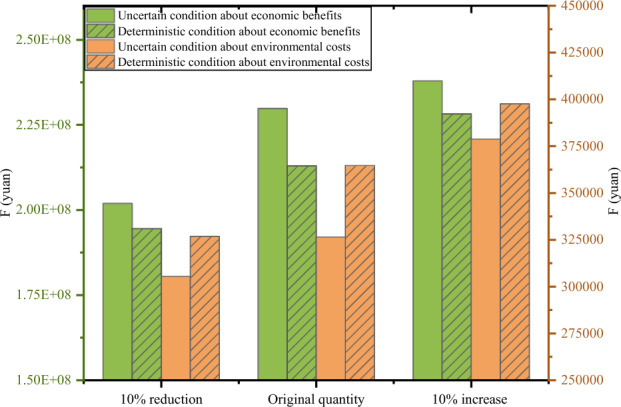
Comparison of economic benefits and environmental in two conditions.

Generally speaking, considering uncertainty in a model can have a substantial impact on the design of a network. After satisfying the constraints, the paper compares uncertain and deterministic conditions. By varying the amount of battery recycling, three scenarios are set up. Figure 6 shows the comparison of the three scenarios in terms of economic benefits and environmental costs under uncertain and deterministic conditions. The deterministic model has about 20,000 kg more carbon emissions than the uncertainty model. The total benefits of the deterministic model are on average 10 million less than those of the uncertain model. It can be observed from the figures that the uncertain condition gives significantly better results than the deterministic condition for all three scenarios, both in terms of economic benefits and environmental costs. Moreover, the difference in economic benefits is distinct between the scenarios.

This indicates that with an increase in the quantity of recycling, the economic benefits will be enhanced, which also encourages companies to focus on battery recycling. The results suggest that uncertainty needs to be taken into account in the model to obtain greater benefits and reduce the impact on the environment. Solutions under uncertainty not only provide greater flexibility for the network but also have a significantly smaller and more meaningful impact on the environment. Region T only represents a fraction of the country. Based on the distribution of registered enterprises for EV battery recycling in China, it is evident that the East and Central regions of China show a greater emphasis on the recycling of EOL batteries. According to the registered enterprise data in Region T which belongs to the East regions, it comprises only 10% of the national total^67^. Additionally, the data from the case study indicates that the quantity of recycling in Region T is approximately 2% of the national total^68^. Based on the findings from the case study in Region T, if the entire nation or even the global community focuses on EOL battery recycling and promotes similar models, it could yield more substantial potential environmental and economic benefits. We recommend the formulation of a nationwide policy for the re-cycling and utilization of EOL batteries, in collaboration with local governments, industries, and various sectors of society, to ensure feasibility and sustainability. This initiative would contribute to a perspective on global sustainable development.

#### Sensitivity analysis

It is clear from the research that many factors influence the total economic benefits of the network, such as the time of recycling, the location of recycling, the quantity of batteries recycled, the quality of the batteries, the unit purchase cost, and relevant policies. This paper considers the uncertainty of the quantity of batteries recycled and the quality of the batteries. Consequently, this section intends to analyze the impact on the model results and the value of the objective function by varying the values of the parameters with uncertainty.

**Figure 7:**
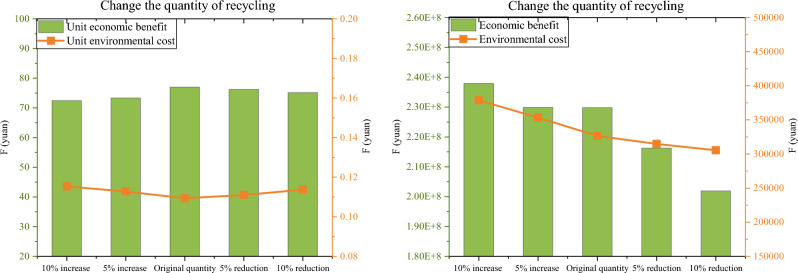
Comparison of results in five scenarios.

Firstly, in order to analyze the changes in the model results, the amount of battery recycled was changed and five scenarios were set up. The results show that the change in recycling quantity has little effect on site selection results. And from Fig. 7, it can be intuitively seen that the difference between the unit economic benefits and unit environmental costs under the five scenarios is also not significant. But due to the scale effect, as the proportion of EOL electric vehicle batteries recycled increases, the total profit of the reverse logistics network tends to increase, so the number of nodal facilities required will also increase. Therefore, the decision maker needs to decide on the number or scale of alternative points for recycling nodal facilities based on the quantity of EOL electric vehicle batteries to be recycled.

**Figure 8:**
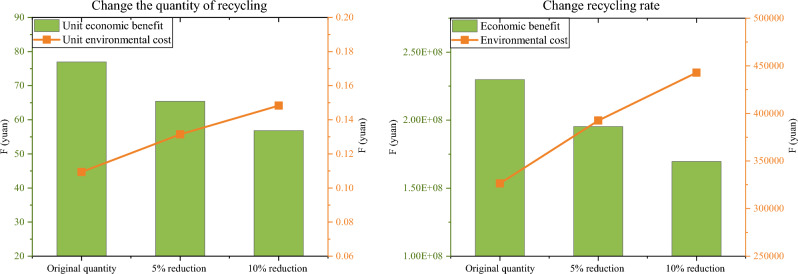
Comparison of results in three scenarios.

Furthermore, the paper also changes the battery recycling rate, similar to changing the recycling quality, which is the probability of moving the battery from the battery processing center to the reuse center. Since the recycling rate in this case is already large enough, this paper analyzes the impact on the network by reducing the remanufacturing recycling rate. As can be seen from Fig. 8, when the recycling rate is reduced by 10%, the total benefit is reduced by 60.205 million yuan and the carbon emission is increased by 116,250 kg. As the recycling rate decreases, the total economic benefits and unit economic benefits decrease significantly, while the environmental costs increase significantly. It is reasonable to assume that an increase in recycling rates should contribute to an increase in total benefits and a decrease in environmental costs. The conclusion suggests that the model results would be better if more EOL batteries were reused. This also proves the advantages of echelon utilization, being more environmentally friendly and sustainable. The lower the quality of the product, the lower the network benefits. Likewise, if the quality of the product is improved, network benefits increase, and then more reuse centers will be needed. Therefore, by comparing the results of the above scenarios, it is found that the changes in recycling rate have a greater impact on the network than the changes in recycling quantity.

## Conclusions

This study introduces an EOL electric vehicle battery recovery network design approach that accounts for uncertainty. In this study, we developed a battery recycling model that incorporates environmental costs and benefits within energy storage scenarios, taking into account the uncertain quality and quantity of EOL batteries. To enhance model clarity, we introduced a stochastic programming approach, combining uncertainty programming and a fuzzy mathematical method. The GA was applied for solving this model. These models and approaches offer a rational decision-making framework for new energy enterprises involved in battery recycling. The results from the Region T case study demonstrate the effectiveness of the proposed method in enhancing network benefits and lowering network costs. In contrast to the deterministic model, the model that accounts for uncertainty is expected to reduce carbon emissions by 20 metric tons and boost overall benefits by approximately 10 million yuan.

Furthermore, the paper concludes that battery processing has the greatest influence on environmental and eco-economic costs. Hence, the primary focus of network optimization is now on developing and implementing cost-effective and environmentally friendly recycling technologies and transportation methods. Moreover, the data above reveals that a 10% reduction in the recycling rate significantly impacts the model's outcomes, leading to a decrease of 60.205 million yuan in total revenue and an increase of 116.25 tons in carbon emissions. This highlights the strong correlation between the recycling rate, economic benefits, and environmental impacts. Increasing the recycling of used batteries not only enhances the likelihood of reuse but also boosts economic benefits, with a reduced negative impact on the environment. Consequently, this study promotes increased active involvement of companies in the recycling of hazardous used batteries. While alterations in recycling volume have a minor influence on the network, they do impact the overall network scale.

To deepen our understanding of EOL battery recycling, further research on uncertainty is necessary. While the uncertainty in the quantity and quality of EOL batteries significantly affects the results of network optimization, it's essential not to overlook other factors, such as collection time and prices, for a more precise establishment of recycling network models. Dealing with uncertainty is crucial to address existing uncertain factors, and methods for handling uncertainty, such as scenario analysis and Monte Carlo simulations, can be applied. Furthermore, there are various optimization algorithms available for model solving. Moreover, future research can compare the impact of different processing and transportation methods on recycling since they have a profound influence on EOL battery recycling.

## Data Availability

The data that support the findings of this study are available on request from the corresponding author. The data are not publicly available due to privacy or ethical restrictions.

## List of symbols

$I = \{ 1,2,...,|I|\} ,i \in I$: Battery collection center
$$M = \{ 1,2,...,|M|\} ,m \in M$$: Reuse center
$J = \{ 1,2,...,|J|\} ,j \in J$: Battery processing center
$N = \{ 1,2,...,|N|\} ,n \in N$: Material recycling center
$Q_{jm}$: The quantity of EOL batteries flowed between battery processing center j and reuse center m
$Q_{jn}$: The quantity of EOL batteries flowed between battery processing center j and material recycling center n
$Q_{ij}$: The quantity of EOL batteries flowed between battery collection center i and battery processing center j
$p_{11}$: Unit economic benefits of energy storage over the life cycle
$p_{n}$: Unit economic benefits of material recycling center n
$g$: Unit economic benefits of government subsidy for recycling of EVB
$d_{ij}$: Distance between battery collection center i and battery processing center j
$d_{jm}$: Distance between battery processing center j and reuse center m
$d_{jn}$: Distance between battery processing center j and material recycling center n
$f_{j}$: Fixed cost of building battery processing center j
$f_{m}$: Fixed cost of building reuse center m
$f_{n}$: Fixed cost of building material recycling center n
$c$: Unit transportation cost between centers
$c_{j}$: The unit processing cost of battery processing center j
$c_{m}$: The unit processing cost of reuse center m
$c_{n}$: The unit processing cost of material recycling center n
$p$: The unit price of battery collection center purchasing EOL batteries
$a$: Unit CO_2_ emissions per distance generated during EOL battery transport
$b$: CO2 emissions from building a center
$e_{j}$: Unit CO_2_ emissions from EOL batteries processing at battery processing center j
$e_{m}$: Unit CO_2_ emissions from EOL batteries processing at reuse center m
$e_{n}$: Unit CO_2_ emissions from EOL batteries processing at material recycling center n
$\beta$: Electric emission factor for energy storage scenario
$$h$$: Carbon tax
$MaxJ$: The upper limit of processing capacity for battery processing center j
$MaxM$: The upper limit of processing capacity for reuse center m
$MaxN$: The upper limit of processing capacity for material recycling center n

## Decision variables

$y_{j} \in \left\{ {0,1} \right\}$: If a battery processing center is established at the location $j \in J$ is 1, otherwise 0
$y_{n} \in \left\{ {0,1} \right\}$: If a material recycling center is established at the location $n \in N$ is 1, otherwise 0
$y_{m} \in \left\{ {0,1} \right\}$: If a reuse center is established at the location $m \in M$ is 1, otherwise 0

## Fuzzy variable

$\tilde{Q}_{i}$: The fuzzy quantity of EOL batteries recycled from battery collection center i
$\tilde{\mu }$: The fuzzy percentage of EOL batteries flowed to reuse center m
$\tilde{\omega }$: The fuzzy percentage of EOL batteries flowed to material recycling center n

## References

[1] Management rules for production access of new energy vehicles. National Development and Reform Commission. http://www.gov.cn/zwgk/2007-10/24/content_785019.htm (2007).

[2] Guo Q, You W (2023). Research on psychological attributions and intervention strategies of new energy hybrid vehicle purchase behavior. Sci. Rep..

[3] Global electric vehicle sales will increase by 55% in 2022. *Ministry of Commerce of the People's Republic of China*. http://tr.mofcom.gov.cn/article/jmxw/202304/20230403406888.shtml (2023).

[4] Li X (2022). Collection mode choice of spent electric vehicle batteries: Considering collection competition and third-party economies of scale. Sci. Rep..

[5] Li Q, Yu XQ, Li H (2022). Batteries: From China's 13th to 14th Five-Year Plan. eTransportation.

[6] Wang L, Wang X, Yang W (2020). Optimal design of electric vehicle battery recycling network–From the perspective of electric vehicle manufacturers. Appl. Energy.

[7] Gu X (2021). Electric vehicle battery secondary use under government subsidy: A closed-loop supply chain perspective. Int. J. Prod. Econ..

[8] https://www.ike-global.com/#/products-2/chinese-lca-database-clcd.

[9] Kilic HS, Cebeci U, Ayhan MB (2015). Reverse logistics system design for the waste of electrical and electronic equipment (WEEE) in Turkey. Resour. Conserv. Recycl..

[10] Alumur SA, Nickel S, Saldanha-da-Gama F, Verter V (2012). Multi-period reverse logistics network design. Eur. J. Oper. Res..

[11] Sasikumar P, Kannan AN (2010). A multi-echelon reverse logistics network design for product recovery—A case of truck tire remanufacturing. Int. J. Adv. Manuf. Technol..

[12] Momenitabar M, Dehdari Ebrahimi Z, Arani M (2022). Designing a sustainable closed-loop supply chain network considering lateral resupply and backup suppliers using fuzzy inference system. Environ. Dev. Sustain..

[13] Al-Salem M, Diabat A, Dalalah D, Alrefaei M (2016). A closed-loop supply chain management problem: Reformulation and piecewise linearization. J. Manuf. Syst..

[14] Yoda S, Ishihara K (1999). The advent of battery-based societies and the global environment in the 21st century. J. Power Sour..

[15] Beer S, Gómez T, Dallinger D, Momber I, Marnay C, Stadler M, Lai J (2012). An economic analysis of used electric vehi-cle batteries integrated into commercial building microgrids. IEEE Trans. Smart Grid.

[16] Kannan G, Sasikumar P, Devika K (2010). A genetic algorithm approach for solving a closed loop supply chain model: A case of battery recycling. Appl. Math. Modell..

[17] Tadaros M (2022). Location of facilities and network design for reverse logistics of lithium-ion batteries in Sweden. Oper. Res..

[18] Ren Y (2023). Hidden delays of climate mitigation benefits in the race for electric vehicle deployment. Nat. Commun..

[19] Kim HC (2016). Cradle-to-gate emissions from a commercial electric vehicle Li-ion battery: a comparative analysis. Environ. Sci. Technol..

[20] Xiong S, Ji J, Ma X (2020). Environmental and economic evaluation of remanufacturing lithium-ion batteries from electric vehicles. Waste Manag..

[21] Hao H (2017). Impact of recycling on energy consumption and greenhouse gas emissions from electric vehicle production: The China 2025 case. Resour. Conserv. Recycl..

[22] Chen Y-W, Wang L-C, Wang A, Chen T-L (2017). A particle swarm approach for optimizing a multi-stage closed loop supply chain for the solar cell industry. Robot. Comput.-Integr. Manuf..

[23] Wang B, Hao H, Li H (2022). Designing a reverse logistics network model for waste batteries. Proc. Bus. Econ. Stud..

[24] Momenitabar M, Ebrahimi ZD, Ghasemi P (2022). Designing a sustainable bioethanol supply chain net-work: A combination of machine learning and meta-heuristic algorithms. Ind. Crops Prod..

[25] Lee DH, Dong M (2009). Dynamic network design for reverse logistics operations under uncertainty. Transp. Res. E Logist. Transp. Rev..

[26] Yadollahinia M, Teimoury E, Paydar M (2018). Tire forward and reverse supply chain design considering customer relationship management. Resour. Conserv. Recycl..

[27] Wang J, Cevik M, Amin SH, Parsaee AA (2021). Mixed-integer linear programming models for the paint waste management problem. Transp. Res. E Logist. Transp. Rev..

[28] Amin SH, Zhang G, Akhtar P (2017). Effects of uncertainty on a tire closed-loop supply chain net-work. Expert Syst. Appl..

[29] Bao JF (2023). Design of reverse logistics network for power battery recycling of retired vehicles considering risk loss. Logist. Technol..

[30] Jafari HR, Abharian AK (2020). Sustainable closed-loop supply chain design for the car battery industry with taking into consideration the correlated criteria for supplier selection and uncertainty conditions. Revista Gestão Tecnologia.

[31] Liang TF (2011). Application of fuzzy sets to manufacturing/distribution planning decisions in supply chains. Inform. Sci..

[32] Hasanov P, Jaber MY, Tahirov N (2019). Four-level closed loop supply chain with remanufacturing. Appl. Math. Modell..

[33] Kammammettu S, Li Z (2023). Scenario reduction and scenario tree generation for stochastic programming using sinkhorn distance. Comput. Chem. Eng..

[34] Dantzig GB (1955). Linear programming under uncertainty. Manag. Sci..

[35] Birge J. R, Louveaux F. Introduction to stochastic programming. *Springer Science & Business Media*. http://www.springer.com/series/3182 (2011).

[36] Cusenza MA (2019). Reuse of electric vehicle batteries in buildings: An integrated load match analysis and life cycle assessment approach. Energy Build..

[37] Kamath D (2020). Evaluating the cost and carbon footprint of second-life electric vehicle batteries in residential and utility-level applications. Waste Manag..

[38] Hu S et al. Assessment of the Economic Value of the Energy Storage Battery Systems. *Journal of Shanghai University of Electric Power* 29: 315–320. https://d.wanfangdata.com.cn/periodical/CiFQZXJpb2RpY2FsQ0hJTmV3UzIwMjIxMDI0MjAyMjEwMjQSEXNoZGx4eXhiMjAxMzA0MDAzGgh5eHA2OTRlMw%3D%3D (2013).

[39] Yang J, Weil M, Gu F (2022). Environmental-economic analysis of the secondary use of electric vehicle batteries in the load shifting of communication base stations: A case study in China. J. Energy Storage.

[40] Hu Y, Armada M, Sánchez MJ (2022). Potential utilization of battery energy storage systems (BESS) in the major European electricity markets. Appl. Energy.

[41] Xu Z (2019). The design of green supply chains under carbon policies: A literature review of quantitative models. Sustainability.

[42] Konstantaras I, Skouri K, Benkherouf L (2021). Optimizing inventory decisions for a closed–loop supply chain model under a carbon tax regulatory mechanism. Int. J. Prod. Econ..

[43] Fattahi R, Khalilzadeh M (2018). Risk evaluation using a novel hybrid method based on FMEA, extended MULTIMOORA, and AHP methods under fuzzy environment. Saf. Sci..

[44] Chen Y, Li B (2011). Dynamic multi-attribute decision making model based on triangular intuitionistic fuzzy numbers. Sci. Iran. B.

[45] Liu B, Iwamura K (1998). Chance constrained programming with fuzzy parameters. Fuzzy Sets Syst..

[46] Wang J, Dan Ding Ou, Liu ML (2016). A synthetic method for knowledge management performance evaluation based on triangular fuzzy number and group support systems. Appl. Soft Comput..

[47] Chang H-C, Yao J-S, Ouyang L-Y (2004). Fuzzy mixture inventory model with variable lead-time based on probabilistic fuzzy set and triangular fuzzy number. Math. Comput. Modell..

[48] Qingda G, Yanming Q, Peijie L, Jianwu C (2018). Trajectory planning of robot based on quantum genetic algorithm. Recent Dev. Mechatron. Intell. Robot..

[49] Amal L, Son L. H, Chabchoub H. SGA: spatial GIS-based genetic algorithm for route optimization of municipal solid waste collection. *Environmental Science and Pollution Research* 25: 27569–27582. https://www.webofscience.com/wos/alldb/full-record/WOS:000444202800079 (2018). 10.1007/s11356-018-2826-0 30054836

[50] Zhang YZ, Zhang ZW. Solving multi-station refuse collection problem based on cooperative co-evolutionary algorithm. *Journal of University of Science and Technology of China* 50(5): 695–704. https://dr2am.wust.edu.cn/--/cn/com/wanfangdata/d/hs/_/periodical/ChlQZXJpb2RpY2FsQ0hJTmV3UzIwMjMwMzIxEhN6Z2t4anNkeHhiMjAyMDA1MDE4GghhMjRlZzg4NQ (2020).

[51] Reeves CR (1993). Modern Heuristic Techniques for Combinatorial Problems.

[52] Whitley D, Starkweather T, Shaner D (1991). The Traveling Salesman and Sequence Scheduling: Quality Solutions Using Genetic Edge Recombination.

[53] http://www.jiangsu.gov.cn/art/2023/2/16/art_60085_10752266.html.

[54] Zhang XT, Xu L (2023). Research and industrialization status of recycling of waste lithium iron phosphate batteries. Multi-Purp. Util. Miner. Resour..

[55] Wang S. Research on Logistics Network Model of Electric Vehicle Waste Battery. *School of Economics and Management*. https://d.wanfangdata.com.cn/thesis/ChJUaGVzaXNOZXdTMjAyMjExMTkSCFkzNjIyMTMzGghseGcydThkaw%3D%3D (2019).

[56] Liu ZW. Modeling and Optimization of Electric Vehicle Power Battery Recycling Network Under Uncertain Conditions, School of Economics and Management (2021).

[57] Quan J (2022). Comparative life cycle assessment of LFP and NCM batteries including the secondary use and different recycling technologies. Sci. Total Environ..

[58] "On doing a good job in 2022 enterprises greenhouse gas emissions report management-related key work of the notice" interpretation. *Ministry of Ecology and Environment of the People's Republic of China*. https://www.mee.gov.cn/zcwj/zcjd/202203/t20220315_971493.shtml (2022).

[59] Slater H, De Boer D, Qian G, Wang S. China Carbon Price Survey Report. ICF: Beijing, China (2021).

[60] Ramezani M, Bashiri M, Tavakkoli-Moghaddam R (2013). A new multi-objective stochastic model for a forward/reverse logistic network design with responsiveness and quality level. Appl. Math. Modell..

[61] Jeong YS, Lee SE, Huh JH (2012). Estimation of CO2 emission of apartment buildings due to major construction materials in the Republic of Korea. Energy Build..

[62] Dunn JB (2012). Impact of recycling on cradle-to-gate energy consumption and greenhouse gas emissions of automotive lithium-ion batteries. Environ. Sci. Technol..

[63] Liu C (2019). Recycling of spent lithium-ion batteries in view of lithium recovery: A critical review. J. Clean. Prod..

[64] Liu C, Lin J, Cao H, Zhang Y, Sun Z (2019). Recycling of spent lithium-ion batteries in view of lithium recovery: A critical review. J. Clean. Prod..

[65] Dunn JB, James C, Gaines L, Gallagher K, Dai Q, Kelly JC (2015). Material and energy flows in the production of cathode and anode materials for lithiumion batteries. Acta Chem. Scand..

[66] Dai Q (2019). EverBatt: A closed-loop battery recycling cost and environmental impacts model (No. ANL-19/16). Argonne Natl. Lab..

[67] https://www.chinabaogao.com/baogao/202202/572436.html.

[68] https://www.miit.gov.cn/xwdt/gxdt/ldhd/art/2023/art_643c641ae55849eabd329f8311bc964d.html.

